# MEPrep: A robust pipeline for multi-echo fMRI denoising and preprocessing

**DOI:** 10.1162/IMAG.a.1198

**Published:** 2026-04-06

**Authors:** Zhishun Wang, Feng Liu, Rachel Marsh, Gaurav H. Patel, Jack Grinband

**Affiliations:** The Department of Psychiatry, Vagelos College of Physicians and Surgeons, Columbia University, New York, NY, United States; New York State Psychiatric Institute, New York, NY, United States

**Keywords:** multi-echo functional MRI (ME-fMRI), functional connectome, fMRIPrep, tedana, independent component analysis (ICA), Nipype

## Abstract

Multi-echo fMRI has emerged as a powerful strategy to mitigate head motion-related noise and minimize susceptibility-related signal loss in BOLD data. Multi-echo independent component analysis (ME-ICA) effectively distinguishes between BOLD-related (TE-dependent) signals and non-BOLD (TE-independent) noise, yielding substantial enhancements in performance compared to traditional echo-combination methods. We introduce a novel ICA-based denoising step, preICA, applied to raw multi-echo data before optimal T2*-weighted echo combination. This approach, combined with ME-ICA, yields substantial gains in data denoising. Our results show that preICA significantly enhances the efficacy of optimal echo combination and ME-ICA to reduce noise. To facilitate the reliable processing of multi-echo fMRI data, we integrated preICA and ME-ICA into fMRIPrep, resulting in the creation of a robust multi-echo processing pipeline, called *MEPrep,* offering flexibility in preprocessing options (with or without preICA and/or ME-ICA) beyond the echo combination approach offered by fMRIPrep. We validated *MEPrep* on an open resting-state multi-echo fMRI dataset, demonstrating that incorporating the preICA step leads to statistically significant improvements in denoising efficacy, as evidenced by (1) enhanced T2* exponential model fitting accuracy; (2) reduced motion-related BOLD fluctuations; (3) increased temporal signal-to-noise ratio; (4) improved spatial and temporal reliability of functional connectivity; and (5) increased Shannon entropy. *MEPrep* outperforms existing pipelines by synergistically integrating preICA and ME-ICA, achieving superior noise suppression while preserving the neurobiological complexity of denoised BOLD signals. By automating multi-echo preprocessing within a robust pipeline, *MEPrep* provides a scalable solution for high-quality multi-echo fMRI data preprocessing. The pipeline is openly available, ensuring reproducibility and accessibility for the neuroimaging community.

## 1 Introduction

Functional connectivity analysis based on resting state functional MRI is a widely used approach for mapping intrinsic functional brain networks implicated in cognition and behavior (Contreras et al., 2015; Finn et al., 2015; Gordon et al., 2017; Horn et al., 2014; Sporns et al., 2005; Van Dijk et al., 2010). However, the reliability of functional connectivity analyses is often compromised by temporal and spatial confounds in the fMRI data (Bright et al., 2017; Liu, 2016; Parkes et al., 2018; Power, 2017; Power et al., 2014, 2015, 2017). For example, the quality of resting-state or task-based fMRI data can be severely impacted by temporal noise from sources like head motion (Van Dijk et al., 2012) and physiological processes such as respiration and cardiac fluctuations (Birn et al., 2006). These noise sources are known to introduce spurious correlations in functional connectivity analyses (Power et al., 2012). In addition, magnetic susceptibility can cause substantial signal loss in brain regions, including orbitofrontal cortex, ventral temporal cortex, and ventral striatum, leading to spatial artifacts (Deichmann et al., 2003). Furthermore, these temporal and spatial components may interact nonlinearly, further degrading fMRI data (Power et al., 2014).

Multi-echo fMRI (ME-fMRI) has emerged as a promising alternative to traditional single-echo fMRI for studying intrinsic brain networks (Baek et al., 2017; Cohen et al., 2017, 2018; Dipasquale et al., 2017; DuPre et al., 2016; Evans et al., 2015; Kundu et al., 2012, 2013, 2015, 2017, 2018; Lynch et al., 2020; Power et al., 2018; Spreng et al., 2019, 2022; Steel et al., 2022; Waltmann et al., 2019). The generation of blood oxygenation level–dependent (BOLD) signal in fMRI follows an exponential decay law governed by echo time (TE) and the tissue-specific relaxation time constant T2*. Here, T2* is critical because changes in tissue oxygenation alter local magnetic field inhomogeneities, thereby modulating T2*. Unlike single-echo fMRI, multi-echo fMRI acquires data at multiple TEs, uniquely enabling direct voxel-wise estimation of T2* by fitting an exponential decay curve across echoes. This capability provides two key advantages: (a) enhanced separation of BOLD signals from noise because TE-dependent BOLD fluctuations linked to T2* are distinguishable from TE-independent noise and (b) optimal combination of echoes by computing a weighted mean according to their T2*-dependent BOLD sensitivity, which maximizes temporal signal-to-noise ratio (tSNR) and improves functional contrast. Recent advancements in pulse-sequence technology, including multi-band imaging (Feinberg et al., 2010; Moeller et al., 2010) available in widely-used scanners from GE and Siemens, have rendered multi-echo acquisitions feasible without compromising spatial or temporal resolution. To fully exploit the potential of ME-fMRI, Kundu and colleagues introduced multi-echo independent component analysis (ME-ICA) (Kundu et al., 2012, 2013, 2015, 2017, 2018), accompanied by the development of a software package (*tedana*) for streamlined ME-ICA implementation (DuPre et al., 2021). Tedana performs T2*-weighted echo combination followed by ICA decomposition to isolate BOLD-related signal components. This method has been successfully applied across a wide range of conditions and phenotypic variation including neurodevelopmental variation (Kundu et al., 2015), ADHD (Dipasquale et al., 2017), and obesity (Baek et al., 2017), resulting in improved functional connectivity analyses through reduction of both Gaussian and non-Gaussian noise (Cohen et al., 2017, 2018; Evans et al., 2015; Spreng et al., 2019, 2022; Steel et al., 2022).

Despite its advantages, tedana exhibits two key limitations. First, its performance depends critically on the quality of the raw multi-echo data, as ME-ICA relies on accurate voxel-wise T2* model fitting and echo-time-dependent metrics to distinguish BOLD-related components from noise (DuPre et al., 2021; Kundu et al., 2012, 2013). Head motion and physiological artifacts can compromise the T2* model fitting and subsequent ICA component classification. Second, tedana is not an end-to-end pipeline since it depends on external preprocessing and downstream processing procedures to generate standard functional imaging outputs ready for statistical analyses. For example, it does not natively accept BIDS-formatted inputs, nor does it support essential steps such as slice-timing correction, motion realignment, spatial normalization, or surface projection. Although scripts like Auto_tedana.py (Dennison, 2025) are available to run tedana on fMRIPrep outputs, they require separate execution of each tool and do not form a truly integrated, BIDS-native preprocessing pipeline. More importantly, such scripts are limited to the ME-ICA step and do not provide downstream processing, including spatial normalization or CIFTI surface generation. Moreover, any integration of tedana with other tools, such as fMRIPrep (Esteban et al., 2019, 2020), requires users to assemble their own custom workflows, potentially introducing inconsistencies in the implementation of preprocessing pipelines across studies.

To address these limitations, we developed *MEPrep*, a robust and fully integrated ME-fMRI preprocessing pipeline. *MEPrep* incorporates a novel pre-denoising step, *preICA*, which applies probabilistic ICA (Beckmann & Smith, 2004) to raw multi-echo data prior to echo combination. By reducing the error in T2* model fitting, *MEPrep* improves the effectiveness of all subsequent procedures. Furthermore, *MEPrep* is built within the fMRIPrep ecosystem, ensuring full BIDS compatibility and reproducibility. It supports four output options reflecting combinations of pre-denoising and ME-ICA processing, each available in volumetric (NIfTI) and surface (CIFTI) formats to facilitate comparisons with historical results. Our validation study, conducted on a public multi-echo resting-state fMRI dataset, illustrates the superiority of utilizing the combination of preICA and ME-ICA across various performance metrics. These metrics included T2* model fitting residuals, DVARS (the spatial standard deviation of consecutive image differences), temporal signal-to-noise ratio of the processed data in volumetric and surface spaces, spatial and dynamical reliability of functional connectivity, and Shannon entropy to characterize data complexity. Together, these developments position *MEPrep* as a fully integrated, BIDS-compatible preprocessing pipeline that enables robust, reproducible, and scalable analysis of multi-echo fMRI data.

## 2 Methods

### 2.1 The framework and implementation of *MEPrep*

**Fig. 1.**
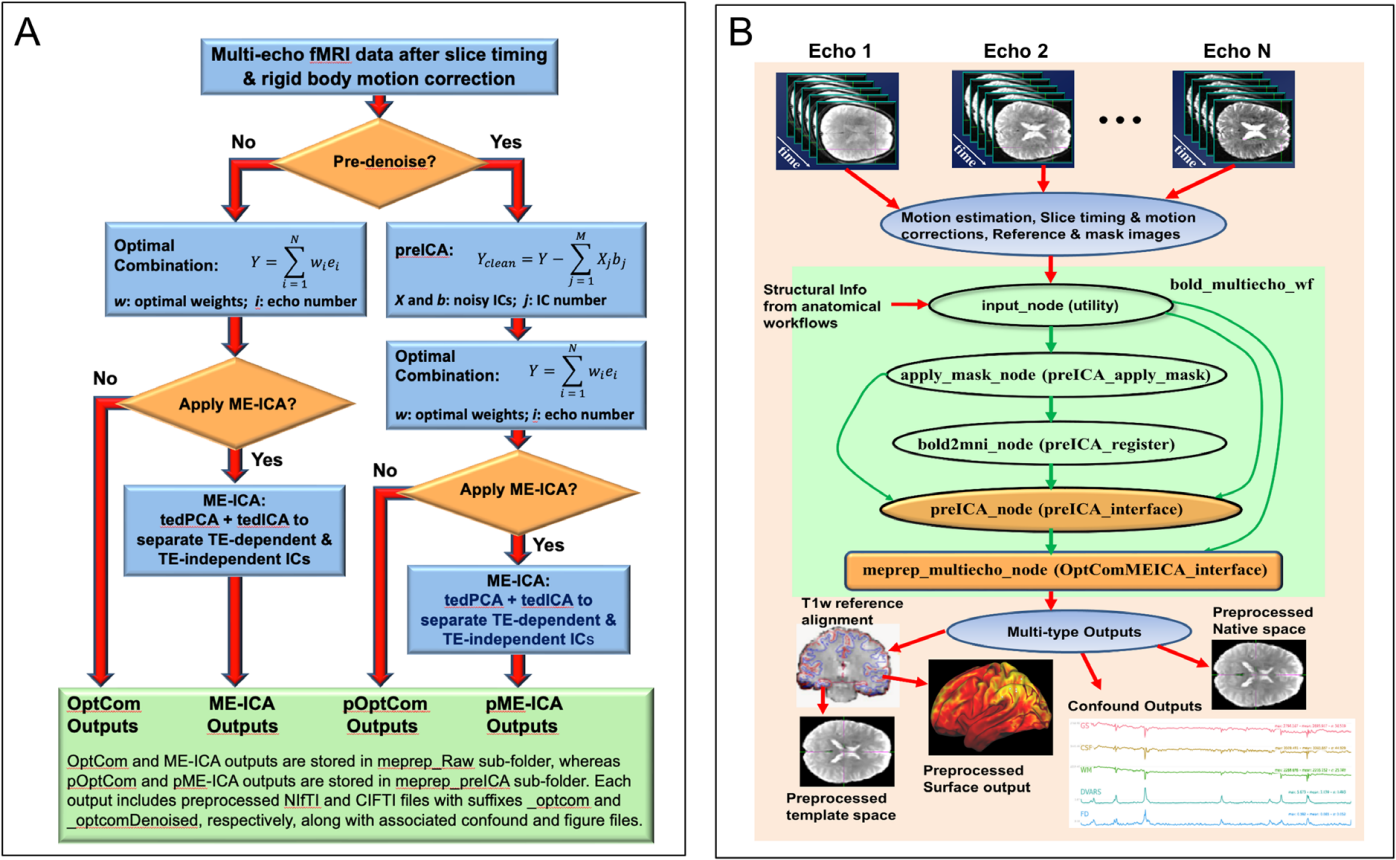
Overview of the *MEPrep* conceptual framework and *Nipype*-based implementation workflows for multi-echo fMRI preprocessing. (A) The conceptual pipeline flowchart outlines the two key decisions in *MEPrep*’s preprocessing workflow: whether to apply ICA-based pre-denoising (preICA) and whether to apply multi-echo independent component analysis (ME-ICA) via tedana. Four resulting output types are generated depending on the chosen path: OptCom Outputs – optimal combination of multi-echo data without preICA or ME-ICA; ME-ICA Outputs – ME-ICA applied to the OptCom output; pOptCom Outputs – pre-denoising via preICA before optimal echo combination; pME-ICA Outputs – ME-ICA applied to pOptCom data, incorporating both preICA and ME-ICA. Each preprocessing path culminates in outputs accompanied by corresponding confound regressors and quality control figures, available in both NIfTI and CIFTI formats. (B) The implementation workflow, developed on the *Nipype* platform, demonstrates how raw multi-echo fMRI data (Echo 1 to Echo N) are processed through modular nodes comprising motion correction, anatomical alignment, and optional preICA denoising. The main bold_multiecho_wf pipeline workflow integrates structural references and applies appropriate transformations, leading to the final meprep_multiecho_wf workflow, which generates outputs in surface space, native volume space, and template space. Confound time series (e.g., FD, DVARS, WM, CSF) are also generated for downstream statistical analyses. This automated, flexible pipeline supports robust, reproducible preprocessing tailored to multi-echo fMRI data.

Figure 1 provides an overview of *MEPrep’s* conceptual framework and its implementation workflows on Nipype platform (Gorgolewski et al., 2011). Figure 1A illustrates the four multi-echo preprocessing strategies it implements, determined by the inclusion of pre-denoising (preICA) and ME-ICA. Specifically, MEPrep generates four corresponding sets of outputs, each produced in both NIfTI and CIFTI formats and accompanied by confound files: (1) OptCom (optimal combination of the multi-echo data); (2) ME-ICA (*tedana* ME-ICA applied to the OptCom outputs); (3) pOptCom (preICA denoising prior to OptCom); and (4) pME-ICA (*tedana* ME-ICA applied to the pOptCom outputs).

*MEPrep* leverages Nipype’s workflow features, including MapNode and iterables, to parallelize preprocessing streams, enabling concurrent execution of preICA and subsequent procedures with and without ME-ICA. This parallelized structure maximizes computational efficiency while allowing direct comparison across denoising strategies without requiring multiple pipeline runs. *MEPrep* integrates established neuroimaging tools within a unified workflow (Fig. 1B): including Nipype (Gorgolewski et al., 2011), fMRIPrep (Esteban et al., 2019), *tedana* (DuPre et al., 2021), FSL (Beckmann & Smith, 2004; Jenkinson et al., 2012), freesurfer (Dale et al., 1999; Fischl et al., 1999), ANTs (Tustison et al., 2014), and ICA-AROMA (Pruim, Mennes, van Rooij, et al., 2015). These components are integrated within the fMRIPrep architecture, leveraging Nipype’s execution model to ensure reproducibility, modularity, and compatibility with future software updates. Additional implementation details are provided in the Supplementary Methods and Supplementary Figures S1–S2.

### 2.2 Pre-denoising multi-echo fMRI data with probabilistic ICA

MEPrep introduces a robust early-stage ICA-based denoising step, termed *preICA*, designed to mitigate motion-related artifacts prior to echo combination. preICA applies the Probabilistic ICA (PICA) algorithm implemented in FSL’s MELODIC toolbox (Beckmann & Smith, 2004). PICA incorporates Bayesian dimensionality estimation, probabilistic principal component analysis, and an expectation–maximization framework, providing improved decomposition stability and reducing sensitivity to overfitting compared with fixed-point ICA approaches such as FastICA, which underlies the tedana ME-ICA implementation.

To accommodate the multi-echo structure, the preICA procedure was performed by applying MELODIC PICA to multi-echo time series that were spatially concatenated along the Z-axis, creating an extended spatial domain while preserving temporal correspondence across echoes. This design provides a robust means of identifying motion-related components that are expressed coherently across echoes, improving the quality of the denoised data prior to subsequent T2*-weighted echo combination and ME-ICA processing.

To identify motion-related components, we extended the validated component-classification framework of ICA-AROMA (Pruim, Mennes, Buitelaar, et al., 2015; Pruim, Mennes, van Rooij, et al., 2015), originally developed for conventional single-echo fMRI, to accommodate the multi-echo data structures generated by the preICA procedure and integrated this extension into the MEPrep pipeline. Briefly, the extended approach identifies motion components by extracting complementary temporal and spatial features from the preICA-derived multi-echo ICA decomposition, including the shared component time-course matrix and the corresponding spatial Z-score maps. We computed two temporal features: the maximum realignment-parameter correlation (maxRPcorr) and the proportion of high frequency content (HFC). Consistent with the original ICA-AROMA framework, maxRPcorr was calculated as follows. A motion model comprising 36 regressors was constructed from the estimated rigid-body motion parameters, including the six original parameters, their temporal derivatives, and their one TR forward- and backward-shifted versions. For each ICA component, Pearson correlations were computed between the component time course and all the 36 motion regressors, as well as between the squared component time course and the squared motion regressors, yielding a total of 72 correlation values per component. The maximum absolute correlation across these values was taken as the motion-sensitivity measure for that component. To obtain a robust estimate, this procedure was repeated over 1,000 random subsamples comprising 90% of the time points, and the final maxRPcorr value was defined as the mean of the maximum correlations across subsamples. Spatial features are derived from suprathreshold component maps, including the edge fraction and cerebrospinal fluid (CSF) fraction, which quantify spatial patterns commonly associated with motion artifacts. In our multi-echo extension, these spatial features are extracted specifically from the first-echo Z-score maps, which provide the highest signal-to-noise ratio among echoes, yielding more robust and reliable estimation of motion-related spatial characteristics. Consistent with the original ICA-AROMA framework, a pretrained linear discriminant analysis (LDA) classifier combines maxRPcorr and edge fraction to identify motion components, while HFC and CSF fraction provide complementary criteria for detecting additional motion-related components. Motion-related components identified by the multi-echo ICA-AROMA extension are regressed out from each individual echo prior to downstream preprocessing.

Compared to the original single-echo version, our multi-echo extension of ICA-AROMA offers several advantages for multi-echo data analysis. First, although the dimensionality of the ICA time-course matrix remains unchanged, the component time courses are derived from spatially concatenated multi-echo data and therefore incorporate shared information across echoes, including covariance of neural signals across different echo times. Consequently, temporal features such as maxRPcorr and HFC, which are computed from the ICA time courses, may be estimated more robustly than in the single-echo formulation. Second, among all echoes, the first echo typically exhibits the highest signal-to-noise ratio. Using the first-echo spatial Z-score map to compute spatial features, such as edge and CSF fractions, provides a more stable and reliable representation of motion-related spatial patterns, thereby improving the robustness of component classification.

### 2.3 Dataset for validations

We validated MEPrep using a publicly available resting-state multi-echo fMRI dataset acquired from 32 healthy participants (mean age 33 ± 13 years; equal gender distribution), as described previously (Kundu et al., 2013). We downloaded this dataset from OpenfMRI (http://www.openfmri.org, #ds000258 (Kundu et al., 2013; Power et al., 2018)). Each participant contributed one resting-state run acquired on a Siemens Trio 3T MRI scanner using a 4-echo EPI sequence (TEs = 12, 28, 44, and 60 ms; TR = 2.47 s; 3.75 mm isotropic voxels). Each run consisted of 239 volumes with a total imaging time of ~590s (2.47*239) or ~10 min (see Supplementary Methods for full acquisition parameters).

### 2.4 Validation metrics and statistical analysis

We compared the four outputs of MEPrep (i.e. OptCom, ME-ICA, pOptCom, and pME-ICA) on six key metrics:

1. Residual of T2* Exponential Model Fitting. We modeled multi-echo BOLD signal decay as:
  $$Y_{BOLD}(t,v,e)\approx S_{0}\left(v\right)\text{exp}(T_{e}\text{​}/\;T_{2}^{*})$$
  and estimated root mean squared error (RMSE) per voxel as:
  $$RMSE(v)=\sqrt{\frac{1}{T\cdot E}\sum_{t=1}^{T}\sum_{e=1}^{E}{\left[Y_{BOLD}\left(t,v,e\right)-S_{0}\text{exp}\left(\frac{T_{e}}{T_{2}^{*}}\right)\right]}^{2}}$$
  where for each time point (*t*), voxel (*v*), and echo (*e*), $S_{0}$ is the intercept, $\;T_{e}$ is the echo time, and $T_{2}^{*}$ is the relaxation time.
2. *DVARS and Carpet Plots.* We computed the spatial standard deviation of temporal derivatives (DVARS) (Power et al., 2014) and visualized them with carpet plots. To enable statistical comparisons of DVARS, we calculated temporal mean of DVARS. We also applied trapezoidal numerical integration to compute the area under the curve (AUC) of DVARS for statistical comparisons.
3. *Temporal Signal-to-Noise Ratio (tSNR).* We computed tSNR for both volumetric NIfTI and surface cortex CIFTI data processed with the four methods in our *MEPrep* pipeline, using the standard formula (Triantafyllou et al., 2006):
  $$tSNR(x)=\;\frac{\bar{x}}{std(x)}$$
  where *x* represents each time series within each voxel or parcel. We then compared the tSNRs between the methods using paired t-test.
4. *Spatial Reliability of Functional Connectivity*. To evaluate the performance of each *MEPrep* method in terms of BOLD data quality, we assessed whether the preprocessed data would generate ROI-based functional connectivity (FC) maps that were better spatially aligned with known networks. We used the networks-32 atlas from the CONN toolbox (Chai et al., 2012; Whitfield-Gabrieli & Nieto-Castanon, 2012), which defines seven major resting-state networks, each serving as a comparison ROI. Spatial reliability was assessed using Pearson correlation between each subject’s FC map and the canonical ROI template map. Cronbach’s alpha (Cronbach, 1951) was computed across networks to assess consistency across subjects, with paired t-tests to compare methods.
5. *Temporal Reliability of Functional Connectivity.* We derived functional connectome matrices from the MEPrep-preprocessed BOLD data in both CIFTI (cortical surface) and NIfTI (volumetric) formats. For the CIFTI data, we employed the Schaefer-300 parcellation atlas with 300 nodes generated via a data-driven approach from the resting-state fMRI data (Schaefer et al., 2018; Yeo et al., 2011). To visualize parcel-wise connectome matrices, we generated CIFTI files in the HCP .pconn.nii format (Van Essen et al., 2012), enabling HCP Workbench to simultaneously display spatial and temporal patterns of functional networks represented by these matrices. For the NIfTI data, we used two volumetric atlas, Networks-32 (Chai et al., 2012; Whitfield-Gabrieli & Nieto-Castanon, 2012) and Networks-268 (Finn et al., 2015). We assessed the temporal reliability of functional connectome by plotting and quantifying test-retest reliability curves over time. Specifically, we computed the Intraclass Correlation Coefficient (ICC) of functional connectome matrix patterns between sliding-windowed segments (partial data) and the full data. This resulted in 20 ICC values corresponding to window lengths ranging from 5% to 100%, with a 5% interval. Plotting these ICC values along the window lengths in ascending order produced a curve that indicates how quickly the similarity reaches a given threshold, such as 95% of the connectome patterns between the partial and full data. This approach allowed us to quantify how consistently the connectivity patterns were preserved across time and to evaluate the stability of our functional connectivity measures.
  To quantify the test-retest reliability curve using a single, interpretable metric, we computed the AUC of the ICC. The AUC-ICC metric provides an interpretable summary of functional connectivity reliability across increasing data lengths, with larger AUC values indicating that high reliability is achieved using less data. By integrating ICC values across multiple time points, AUC jointly captures both the magnitude and stability of reliability rather than relying on a single arbitrary threshold. This approach follows established practice in the functional connectivity literature, where AUC-based summaries are commonly used to compare reliability profiles across preprocessing strategies and datasets (Birn et al., 2013; Gordon et al., 2017; Noble et al., 2017; Zuo & Xing, 2014). In addition, we quantified the minimum percentage (MP) of data required to reach a predefined reliability threshold, defined as a 95% pattern similarity between functional connectome matrices derived from partial data and those derived from the full time series. The relationship between reliability and amount of data used is monotonically increasing, with higher AUC values and lower MP values indicating greater reliability of functional connectivity estimates.
6. *Shannon Entropy.* Shannon entropy measures the complexity of a time series and can be used to assess the integrity of the information it contains. It has been successfully applied to assessing the complexity of fMRI time series (Akdeniz, 2017; Hull & Morton, 2023; Viol et al., 2017). We employed Shannon entropy to determine whether the increases in tSNRs of the BOLD data processed using the four pipelines were due to reductions in complexity. To minimize interference from confounding brain regions such as white matter and cerebrospinal fluid (CSF), we focused on the processed CIFTI data representing the gray matter surface.

## 3 Results

To evaluate MEPrep’s performance in preprocessing multi-echo fMRI data, we assessed six key metrics across the four pipeline variants: OptCom, pOptCom, ME-ICA, and pME-ICA. Statistical comparisons included paired t-tests for each metric and method, summarized in Table 1.

**Table 1.** A summary of pipeline performance in preprocessing multi-echo fMRI data with and without preICA.

	Pipeline		Performance metrics				
Methods	fMRIPrep	MEPrep	Model fitting residual	Average of DVARS	tSNR	Spatial reliability (CA) of FC^^^	Temporal reliability (AUC, MP) of FC^#^
OptCom	Yes	Yes					
pOptCom	None	Yes	OptCom > pOptCom 22.3 ± 1.0 vs. 15.3 ± 0.7, p < 10^-12^, *d* = 2.1, CI = [5.8, 8.1]	OptCom > pOptCom 19.8 ± 1.5 vs. 10.3 ± 0.6, p < 10^-8^, *d* = 1.4, CI = [7.1, 11.9]	pOptCom > OptCom 305.6 ± 25.4 vs. 173.3 ± 10.3, p < 10^-7^, *d* = 1.3, CI = [96.5, 168.0]	pOptCom > OptCom 0.75 ± 0.007 vs. 0.70 ± 0.007, p < 10^-8^, *d* = 1.5, CI = [0.04, 0.06]	pOptCom > OptCom AUC: 17.3 ± 0.08 vs.16.7 ± 0.1, p < 10^-5^,*d* = 1.0, CI = [0.4, 0.9]; MP:55.0 ± 1.9%vs. 65.5± 2.0%, p < 10^-4^, *d* = -0.9, CI = [-14.9, -6.0]
ME-ICA	None	Yes	ME-ICA = OptCom No preICA applied	OptCom > ME-ICA 19.8 ± 1.5 vs. 13.7 ± 0.6, p < 10^-5^, *d* = 0.93, CI = [3.8, 8.4] ME-ICA > pOptCom 13.7 ± 0.6 vs. 10.3 ± 0.6, p < 10^-11^, *d* = 2.0, CI = [2.8, 4.0]	ME-ICA > OptCom (222.9 ± 12.6 vs. 173.3 ± 10.3, p < 10^-6^, *d* = 1.1, CI = [33.5, 65.6]) pOptCom > ME-ICA 305.6 ± 25.4 vs. 222.9 ± 12.6, p < 10^-4^, *d* = 0.81, CI = [46.1, 119.3]	ME-ICA > OptCom (0.71 ± 0.01 vs. 0.70 ± 0.007, p < 0.03, *d* = 0.42, CI = [0.003, 0.03) pOptCom > ME-ICA (0.75 ± 0.007 vs. 0.71 ± 0.01, p < 10^-4^, *d* = 0.83, CI = [0.02, 0.05]	ME-ICA > OptCom AUC:17.0 ± .0.08 vs. 16.7 ± 0.1, p < 0.02, *d* = 0.5, CI = [0.08, 0.6]; MP:59.2 ± 1.6% vs. 65.5 ± 2.0%, p < 10^-2^, *d* = -0.5, CI = [-10.4, -2.1] pOptCom > ME-ICA AUC: 17.3 ± 0.08 vs.17.0 ± 0.08, p < 10^-4^, *d* = 0.9, CI = [0.2, 0.4]; MP:59.2 ± 1.6% vs. 55.0 ± 1.9%, p < 0.02, *d* = -0.5, CI = [-7.5, -1.0]
pME-ICA	None	Yes	OptCom > pME-ICA 22.3 ± 1.0 vs. 15.3 ± 0.7, p < 10^-12^, *d* = 2.1, CI = [5.8, 8.1]	OptCom > pME-ICA 19.8 ± 1.5 vs. 8.8 ± 0.3, p < 10^-8^, *d* = 1.4, CI = [8.2, 13.8] pOptCom > pME-ICA 10.3 ± 0.6 vs. 8.8 ± 0.3, p < 10^-3^, *d* = 0.74, CI = [0.79, 2.3] ME-ICA > pME-ICA 13.7 ± 0.6 vs. 8.8 ± 0.3, p < 10^-11^, *d* = 2.0, CI = [4.0, 5.8]	pME-ICA > OptCom 345.1 ± 150.1 vs. 173.3 ± 58.4, p < 10^-9^, *d* = 1.5, CI = [131.2, 212.4] pME-ICA > pOptCom 345.1 ± 26.5 vs. 305.6 ± 25.4, p < 10^-4^, d = 0.81, CI = [21.9, 57.1] pME-ICA > ME-ICA 345.1 ± 26.5 vs. 222.9 ± 12.6, p < 10^-6^, *d* = 1.1, CI = [81.9, 162.6]	pME-ICA>OptCom 0.76 ± 0.005 vs. 0.70 ± 0.007, p < 10^-10^, *d* = 1.8, CI = [0.05, 0.08] pME-ICA > OptCom 0.76 ± 0.005 vs. 0.75 ± 0.007, p < 0.01, *d* = 0.50, CI = [0.003, 0.02] pME-ICA > ME-ICA 0.76 ± 0.005 vs. 0.71 ± 0.010, p < 10^-5^, *d* = 1.1, CI = [0.03, 0.06]	pME-ICA > OptCom AUC: 17.4 ± 0.07 vs. 16.7± 0.1, p < 10^-6^, *d* = 1.1, CI = [0.5,1.0]; MP:51.6 ± 1.8%vs. 65.5± 2.0%, p < 10^-6^, *d* =- 1.1, CI = [-18.4, -9.4] pME-ICA > pOptCom AUC:17.4 ± 0.07 vs.17.3± 0.08, p < 10^-3^, *d* = 0.8, CI = [0.06, 0.2]; MP:51.6 ± 1.8%vs. 55.0± 1.9%, p < 10^-3^, *d* = -0.7, CI = [-5.2, -1.6] pME-ICA > ME-ICA AUC: 17.4 ± 0.07 vs. 17.0 ± 0.08, p < 10^-8^, *d* = 1.4, CI = [0.3, 0.5]; MP:51.6 ± 1.8% vs. 59.2 ± 1.6%, p < 10^-5^, *d* = -1.0, CI = [-10.3, -5.0]

Notes: ^^^The mean of spatial reliability of functional connectivity (FC) was assessed using Cronbach’s alpha (CA).

# For the area under the curve (AUC) of the ICC curve, greater AUC indicates more reliable FC. For the minimum percentage of data (MP) required to compute an FC matrix with a pattern similarity reaching 95% compared with the connectome pattern generated using the full data, smaller MP indicates more reliable FC.

*d* = Cohen’s *d*; CI = 95% confidence interval.

### 3.1 Residuals of T2* exponential model fitting

**Fig. 2.**
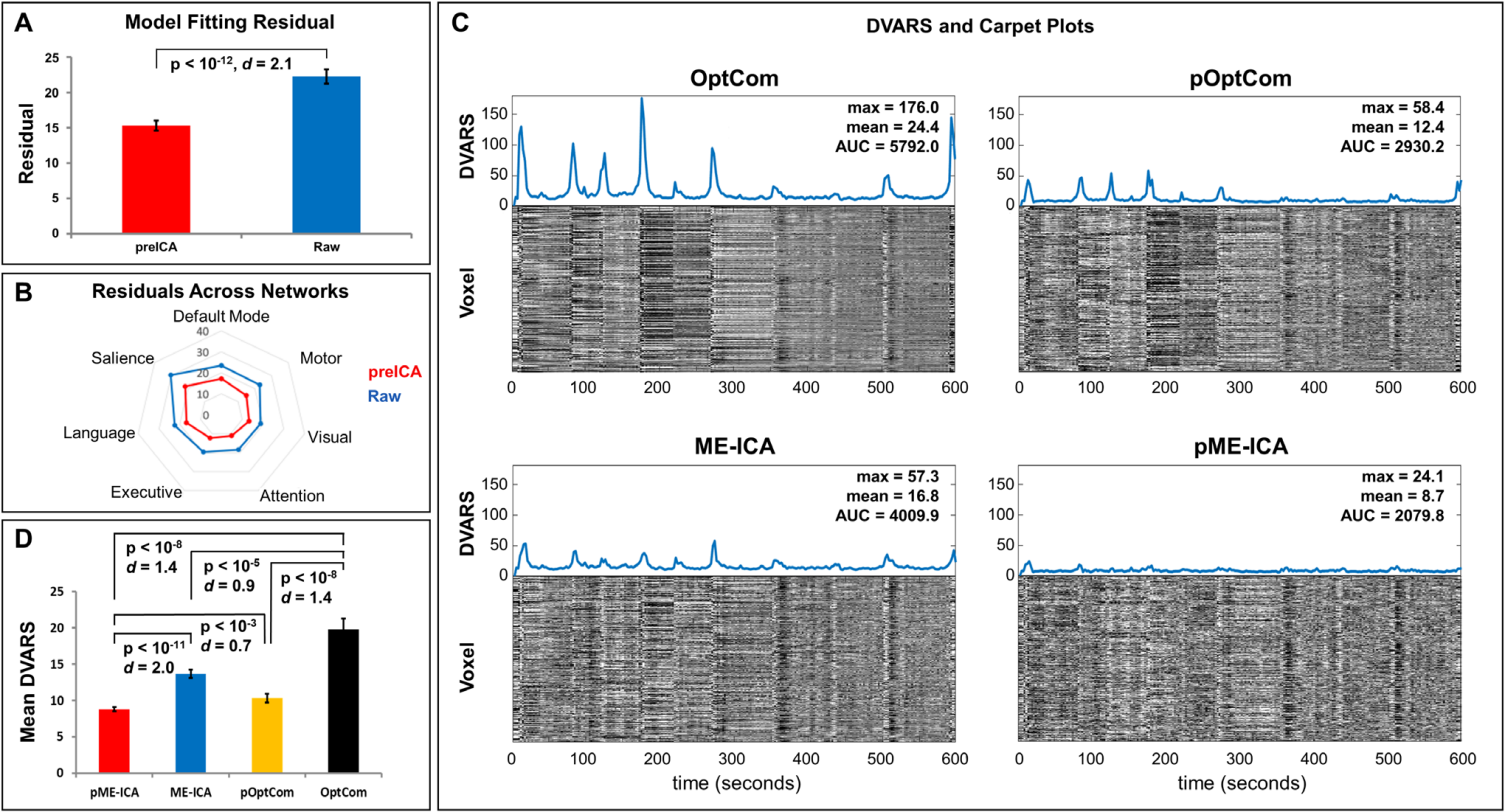
Evaluation of *MEPrep*’s denoising performance through model fitting residuals, DVARS, and carpet plots. (A) Comparison of general linear model fitting residuals between raw multi-echo data and data denoised using preICA. Application of preICA significantly reduces residual variance (p < 10^-12^, Cohen’s *d* = 2.1), suggesting improved model fit. (B) Radar plot showing the distribution of residuals across resting-state networks (Default Mode, Salience, Language, Executive, Attention, Visual, Motor). The red trace (preICA) consistently shows lower residuals than the blue trace (Raw), indicating that preICA enhances signal quality across multiple resting-state functional networks. (C) DVARS and carpet plots for four preprocessing methods. OptCom shows high-amplitude DVARS spikes and pronounced vertical striping patterns in the carpet plot indicate motion-related artifacts; preICA before optimal echo combination, pOptCom, reduces both DVARS amplitude and visible motion artifacts; application of tedana ME-ICA to OptCom data results in further reduction of signal variability and temporal artifacts; combined preICA and ME-ICA approach yields the lowest DVARS amplitude, reflecting the most effective denoising. DVARS metrics (Max, Mean, AUC) are annotated for each condition. (D) Bar plot comparing mean DVARS across the four preprocessing outputs. The pME-ICA method produces the lowest DVARS values, followed by ME-ICA, pOptCom, and OptCom. All pairwise differences are statistically significant (p < 10^-3^ or smaller), indicating cumulative benefits of incorporating both preICA and ME-ICA. Error bars represent standard error of the mean.

preICA produced a substantial reduction in T2* model fitting residuals relative to data without preICA (Fig. 2A; Table 1, p < 10^-12^, Cohen’s *d* = 2.1), reflecting improved fidelity of the exponential decay model. Network-level analyses showed that this improvement was consistent across all the seven large-scale networks (Fig. 2B).

### 3.2 DVARS and carpet plots

Figure 2C presents DVARS time series and carpet plots for a representative subject. Data processed with OptCom retained prominent motion-related artifacts, evidenced by elevated DVARS values and conspicuous banding patterns in the carpet plot. Both pOptCom and ME-ICA substantially attenuated these motion-related features. In contrast, pME-ICA yielded the lowest DVARS values and carpet plots characterized by reduced motion-related stripe patterns, fewer global signal spikes, and smoother frame-to-frame intensity transitions (Waller et al., 2022), indicating the most effective suppression of motion artifacts.

Comparisons across subjects (Table 1; Fig. 2D) confirmed these observations. pOptCom significantly reduced mean DVARS relative to OptCom (p < 10^-8^, Cohen’s *d* = 1.4). ME-ICA also improved over OptCom (p < 10^-8^, Cohen’s *d* = 0.9). Notable, pME-ICA outperformed both ME-ICA (p < 10^-11^, Cohen’s *d* = 2.0) and OptCom (p < 10^-8^, Cohen’s *d* = 1.4).

### 3.3 Temporal signal-to-noise ratio (tSNR)

**Fig. 3.**
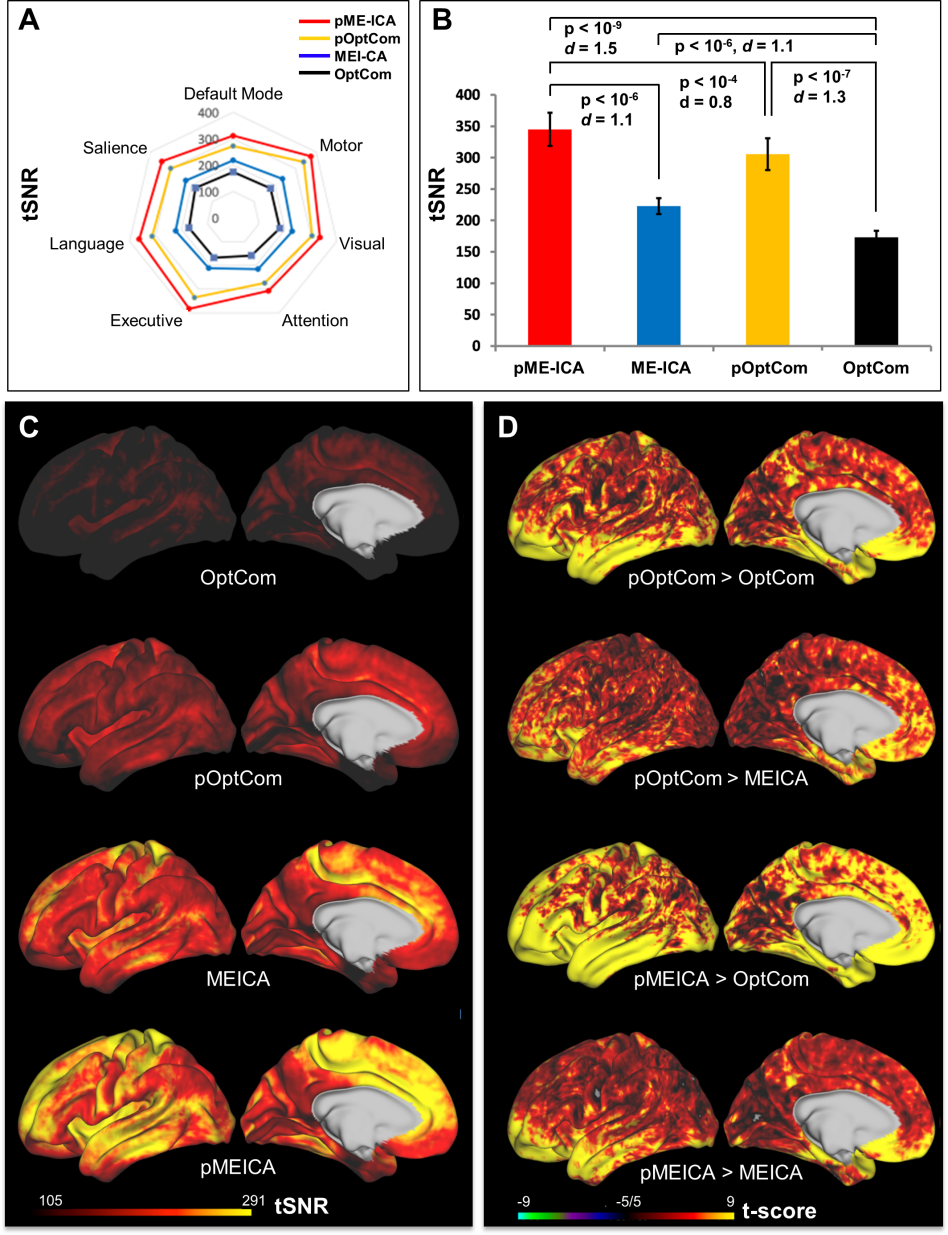
Comparison of temporal signal-to-noise ratio (tSNR) across different *MEPrep* preprocessing pipeline methods. (A) Radar plot of average vertex-wise tSNR across resting-state functional networks for each method: pME-ICA (red), ME-ICA (blue), pOptCom (yellow), and OptCom (black). The pME-ICA method consistently exhibits the highest tSNR across all networks, reflecting superior preservation of BOLD signal quality. (B) Bar plot showing whole-brain mean tSNR for each method. pME-ICA significantly outperforms all others (ME-ICA, p < 10^-6^; pOptCom, p < 10^-4^; OptCom, p < 10^-12^), followed by pOptCom, ME-ICA, and OptCom, confirming cumulative denoising benefits. (C) Cortical surface maps of tSNR for each method, showing widespread enhancement of tSNR from OptCom to pME-ICA. Warmer colors indicate higher signal fidelity. (D) Paired t-score surface maps highlighting statistically significant differences in tSNR between methods. Comparisons show clear gains in tSNR with pOptCom over OptCom, pOptCom over ME-ICA, pME-ICA over OptCom, and pME-ICA over ME-ICA. Yellow regions indicate cortical areas where the denoising method on the left yields significantly higher tSNR than the method on the right (p < 0.05, FDR corrected). These results demonstrate that combining preICA and ME-ICA (pME-ICA) provides the most effective enhancement of temporal SNR in multi-echo fMRI preprocessing.

Table 1 summarizes the results of six possible within-group statistical comparisons of the tSNRs, derived from the multi-echo dataset preprocessed by *MEPrep* across the four methods (OptCom, pOptCom, ME-ICA, and pME-ICA) in volumetric NIfTI format. Radar charts (Fig. 3A) demonstrate that the data preprocessed with pME-ICA show the highest tSNR, while OptCom exhibits the lowest tSNR across all seven large resting-state networks. Notably, pOptCom and ME-ICA outperform OptCom, with pME-ICA surpassing both pOptCom and ME-ICA in improving the tSNR across all networks. Figure 3B confirms these observations; the tSNR (averaged across the networks) was significantly higher for pME-ICA than the other methods.

Figure 3C presents the tSNR amplitudes derived from the whole-brain cortex data for each of the four methods in surface CIFTI format, while Figure 3D displays the results of paired t-tests between methods. Specifically, Figure 3D shows t-score values that survived FDR correction for multiple comparisons, with a stringent threshold of p < 0.001. These figures demonstrate that, similar to the volumetric data, the surface data processed with pME-ICA outperformed OptCom for most of the cortical surface. The pOptCom versus OptCom (first row) and pME-ICA versus ME-ICA (last row) also demonstrates that the preICA step substantially improves tSNR in areas with high susceptibility artifacts such as orbitofrontal and ventral temporal cortex. To quantify the extent of these differences, we calculated the percentage of significantly differing vertexes (p < 0.001, FDR-corrected) over the entire 32k-sampled left and right hemispheres (excluding NaN vertexes) for all six possible pairwise method comparisons. Relative to OptCom, all three alternative methods (pME-ICA, pOptCom, and ME-ICA) yielded significantly higher tSNR in at least 99.96% of cortical vertexes (Table 2). Among these approaches, pME-ICA demonstrated the most extensive improvements, showing higher tSNR in 99.56% of vertexes compared with ME-ICA, 78.52% compared with pOptCom, and 99.99% compared with OptCom.

**Table 2.** Percentage of cortical vertexes showing significant differences in tSNR between methods.

Comparison	% Significant vertexes
pME-ICA vs. ME-ICA	99.56%
pME-ICA vs. pOptCom	78.52%
pME-ICA vs. OptCom	99.99%
pOptCom vs. ME-ICA	79.09%
pOptCom vs. OptCom	99.99%
ME-ICA vs. OptCom	99.96%

### 3.4 Spatial reliability of functional connectivity

**Fig. 4.**
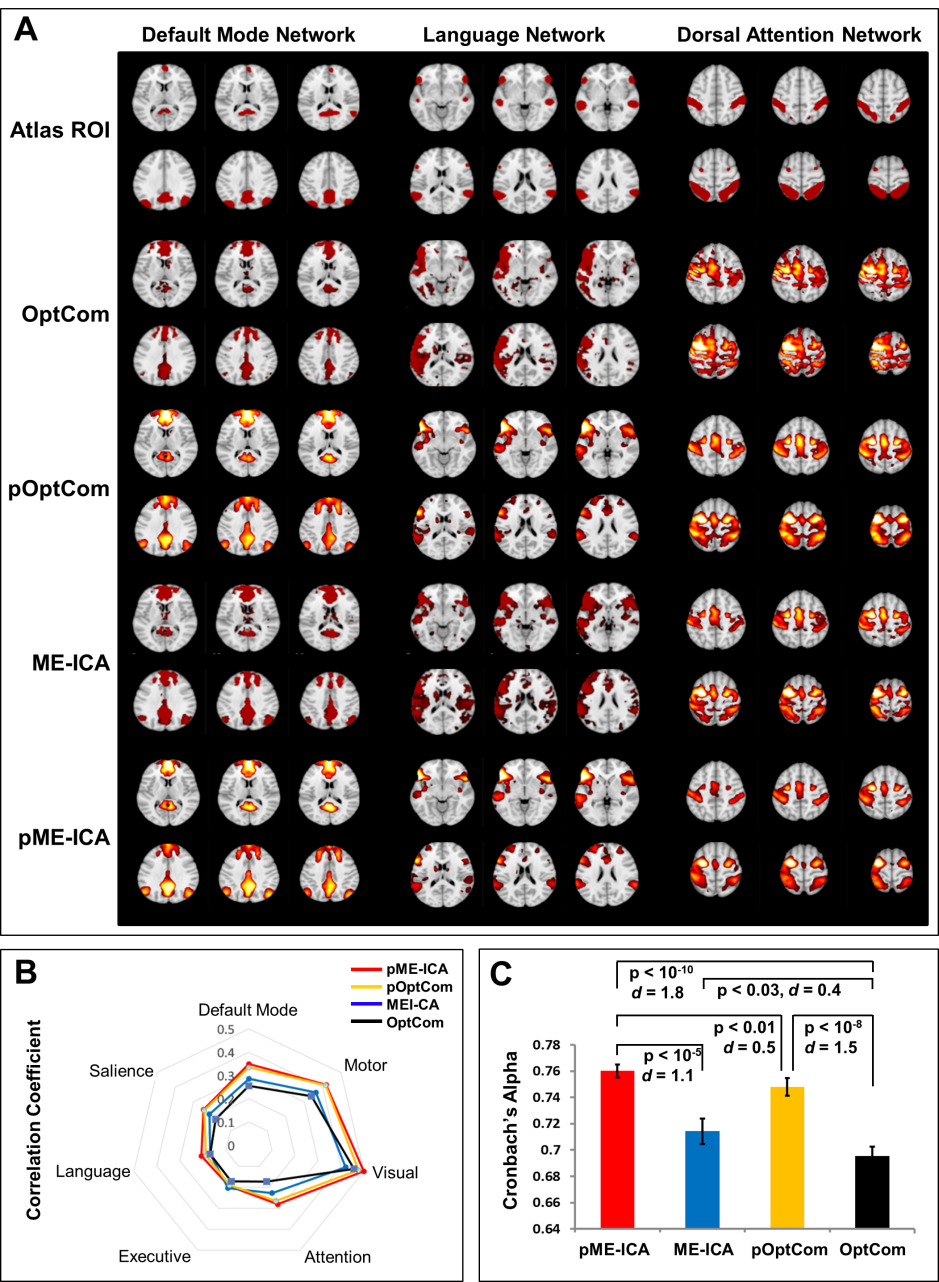
Spatial reliability of ROI-based functional connectivity (FC) maps across *MEPrep* preprocessing methods. (A) Group-level FC maps derived from three representative resting-state networks—the Default Mode Network (DMN), Language Network, and Dorsal Attention Network—are shown for each preprocessing method (OptCom, pOptCom, ME-ICA, and pME-ICA), alongside the corresponding canonical ROI masks from the CONN toolbox’s Networks-32 atlas (top row). Among the methods, preprocessing with pME-ICA produces FC maps that are spatially most aligned with the atlas-defined ROIs, followed by pOptCom and ME-ICA, suggesting improved fidelity in functional connectivity estimation relative to OptCom. (B) Radar plot of mean spatial correlation coefficients between each subject’s FC map and the canonical ROI template across seven major resting-state networks (Default Mode, Salience, Language, Executive, Attention, Visual, and Motor). The pME-ICA method (red) achieves the highest spatial correlations across all networks, followed by pOptCom and ME-ICA, indicating superior preservation of network-level spatial specificity. (C) Bar plot of Cronbach’s alpha coefficients, quantifying cross-subject consistency of FC spatial maps for each method. The pME-ICA pipeline achieves the highest alpha, significantly outperforming ME-ICA (p < 10^-5^), pOptCom (p < 0.01), and OptCom (p < 10^-10^). All pairwise differences are statistically significant (p < 0.03 or smaller), demonstrating that combined preICA and ME-ICA denoising produces the most reliable and reproducible functional connectivity patterns from multi-echo BOLD data.

As summarized in Table 1, preprocessing with preICA significantly improved the spatial reliability of functional connectivity. Figure 4A presents representative mean functional connectivity maps for the default mode, language, and dorsal attention networks, demonstrating stronger spatial correspondence between the connectivity patterns derived from preICA-based methods and their respective network templates. This pattern was consistent across all seven large-scale resting-state networks, as quantified by the radar charts (Fig. 4B). Group-level comparisons of spatial reliability, quantified using Cronbach’s alpha across network-specific functional connectivity maps, are presented in Table 1 and Figure 4C. pME-ICA exhibited significantly higher spatial reliability than all other preprocessing strategies, including OptCom (p < 10^-10^, Cohen’s *d* = 1.8), ME-ICA (p < 10^-5^, Cohen’s *d* = 1.1), and pOptCom (p < 0.01, Cohen’s *d* = 0.5).

### 3.5 Temporal reliability of functional connectivity

**Fig. 5.**
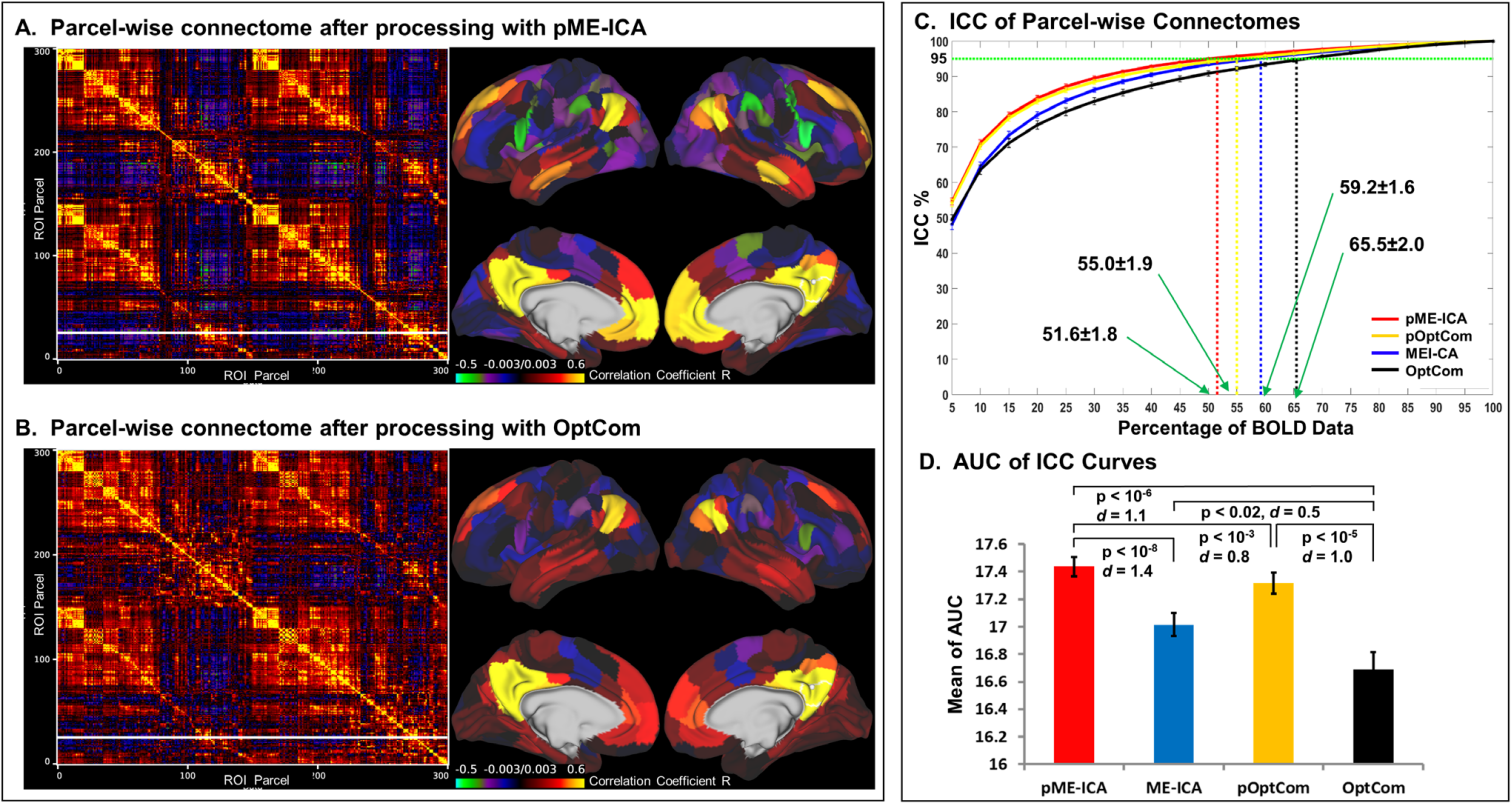
Evaluation of parcel-wise functional connectomes derived from *MEPrep*-preprocessed multi-echo fMRI data. (A) Parcel-wise Functional connectome matrix (based on Schaefer 17-networks atlas with 300 nodes) (left) and cortical parcellation visualization (right) using HCP Workbench from data preprocessed using pME-ICA, which integrates preICA denoising and ME-ICA. The connectome shows enhanced modular organization and inter-network structure, and the corresponding cortical parcellation indicates well-preserved network topography. (B) Connectome matrix and cortical parcellation from data preprocessed with OptCom (optimal echo combination without additional denoising). Compared to pME-ICA, OptCom data exhibit more diffuse connectivity patterns and reduced modular clarity, suggesting residual noise influence. (C) Intraclass correlation coefficient (ICC) curves assessing the test–retest reliability of parcel-wise functional connectomes as a function of the percentage of BOLD data used. The minimum percentage (MP) of data required to reach a reliability threshold of ICC ≥ 0.95 is marked for each method. A lower MP indicates greater reliability with less data. The pME-ICA method (red) achieves the lowest MP (51.7 ± 1.8%), significantly outperforming pOptCom (55.0 ± 1.9%, p < 10^-3^), ME-ICA (59.2 ± 1.6%, p < 10^-5^), and OptCom (65.5 ± 2.0%, p < 10^-6^). All pairwise differences between methods are statistically significant, with pME-ICA consistently requiring the least data to achieve high reliability, demonstrating superior robustness in functional connectome estimation. (D) Mean area under the curve (AUC) of the ICC curves for each preprocessing method. The pME-ICA approach yields the highest AUC, significantly outperforming ME-ICA (p < 10^-8^), pOptCom (p < 10^-3^), and OptCom (p < 10^-6^). These results highlight that combining preICA with ME-ICA denoising improves both the stability and reproducibility of functional connectivity estimates in multi-echo fMRI data.

Figure 5 illustrates the temporal reliability of FC using the CIFTI-based Schaefer-300 parcellation. The pME-ICA method resulted in stronger within-network and weaker between-network connectivity compared to OptCom (Fig. 5A&B). ICC curves (Fig. 5C) demonstrated higher test-retest reliability for preICA methods. Temporal reliability was quantified by two metrics: AUC of ICC curves and the minimum percentage (MP) of data required to achieve ICC ≥ 0.95. Notably, pME-ICA showed an MP that was 21% lower than OptCom and 13% lower than ME-ICA. Both metrics showed a consistent advantage for pME-ICA (Supplementary Fig. S3) in volumetric data using the NIfTI-based Networks-32 and Networks-268 parcellations.

### 3.6 Shannon entropy

**Fig. 6.**
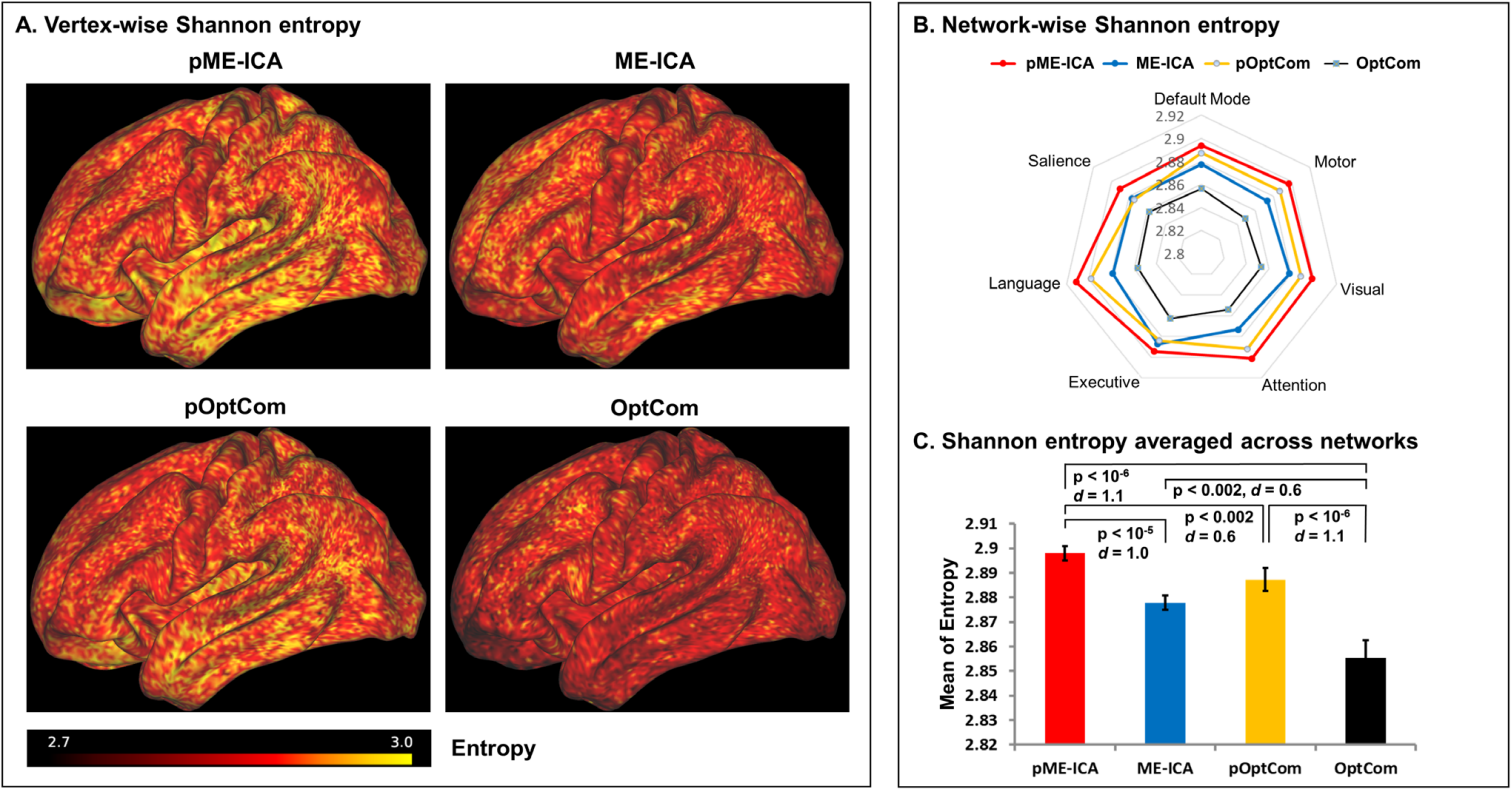
Analysis of Shannon entropy in *MEPrep*-preprocessed multi-echo BOLD data across different denoising methods. (A) Vertex-wise maps of Shannon entropy calculated from BOLD signals for each preprocessing method: pME-ICA, ME-ICA, pOptCom, and OptCom. Warmer colors indicate higher entropy levels, reflecting greater signal complexity and richer temporal dynamics. The pME-ICA map exhibits the most widespread and intense high-entropy regions, suggesting superior preservation of neural signal complexity. (B) Radar plot comparing network-wise mean Shannon entropy across the seven resting-state functional networks (Default Mode, Salience, Language, Executive, Attention, Visual, Motor). The pME-ICA method (red) consistently achieves the highest entropy across all networks, followed by ME-ICA (blue), pOptCom (yellow), and OptCom (black), demonstrating a progressive improvement in preserving information content with more advanced denoising. (C) Bar plot of the average Shannon entropy across the seven major resting-state networks, with statistical comparisons conducted using paired t-tests. The pME-ICA method yields the highest entropy values, significantly outperforming ME-ICA (p < 10^-5^), pOptCom (p < 0.002), and OptCom (p < 10^-6^). These findings suggest that the combined use of preICA and ME-ICA provides the most effective preservation of functional signal complexity in multi-echo BOLD data.

The Shannon entropy metrics were derived from the whole-brain cortex surface data for each of the four MEPrep methods. Figure 6A shows vertex-wise Shannon entropy values (averaged across the 32 subjects). The patterns are similar across methods, with values falling within narrow ranges. Paired t-tests of the entropy metrics did not reveal significant differences between methods (FDR-corrected p value threshold p < 0.05), even though pME-ICA, ME-ICA and pOptCom exhibit slightly higher tSNRs than OptCom. This suggests that the increased tSNRs in pME-ICA, ME-ICA and pOptCom are due to a reduction in noise, particularly motion artifacts, rather than a reduction in complexity or information loss.

Since most resting-state fMRI studies focus on well-established large-scale networks (e.g., default mode and attention networks), we repeated the Shannon entropy comparisons across seven canonical resting-state networks. The resulting network-wise measures, illustrated in the radar plots (Fig. 6B), reveal that pME-ICA consistently achieves the highest entropy across all the seven networks. We also tested the four preprocessing methods using a global measure of entropy, averaged across the seven networks. A one-way repeated-measures ANOVA demonstrated a significant main effect of preprocessing method on entropy (p < 10^−7^), indicating that entropy differed systematically across pipelines. Using paired t-tests, pME-ICA, ME-ICA, and pOptCom all exhibited significantly higher entropy than OptCom. Among these methods, pME-ICA demonstrated the highest overall entropy, exceeding OptCom (p < 10^−6^, Cohen’s *d* = 1.1), ME-ICA (p < 10^−5^, Cohen’s *d* = 1.0) and pOptCom (p < 0.002, Cohen’s *d* = 0.6). All reported differences remained significant after Bonferroni correction (six pairwise comparisons; corrected α = 0.05/6).

## 4 Discussion

This study introduces MEPrep, a fully automated, BIDS-compatible preprocessing pipeline for multi-echo fMRI that integrates early echo-specific denoising, optimized T2*-weighted signal combination, and flexible integration with the fMRIPrep ecosystem. Across all evaluated metrics (tSNR, DVARS, residuals from T2* model fitting, functional connectivity reliability, and Shannon entropy), MEPrep consistently outperformed standard multi-echo pipelines, including OptCom (fMRIPrep) and ME-ICA (tedana). These findings underscore the value of early echo-domain denoising and establish MEPrep as a robust and scalable solution for multi-echo preprocessing. Notably, as expected and consistent with prior reports (Kundu et al., 2012, 2013, 2017), ME-ICA outperformed OptCom, providing an important confirmation of ME-ICA’s known advantages.

### 4.1 Implications for neuroimaging research

The improvements in signal fidelity and test-retest reliability observed with MEPrep have important implications for both basic and clinical fMRI research. Increases in tSNR and reductions in DVARS indicate reduced measurement noise and greater robustness to motion artifacts, two key determinants of statistical power in fMRI analyses. By decreasing random and structured variance in functional connectivity estimates, these improvements are expected to reduce spurious correlations and thereby lower susceptibility to false positive findings (Type I error). At the same time, MEPrep substantially improves test-retest reliability, as reflected by higher ICC values and a 13–21% reduction in the amount of data required to achieve ICC ≥ 0.95 compared to OptCom or ME-ICA alone (Fig. 5, Table 1). Increased reliability enhances the stability of true connectivity signals across time, improving sensitivity to genuine effects and reducing the likelihood of false negatives (Type II error). Together, these properties indicate that MEPrep improves the statistical efficiency of fMRI studies, enabling shorter more tolerable scans for populations with limited scan tolerance, such as children, older adults, and clinical groups.

Equally important are MEPrep’s architectural advantages. Unlike tedana, which lacks support for surface-based outputs and must be manually integrated into existing workflows, MEPrep supports both volumetric and CIFTI outputs and adheres to BIDS conventions. This interoperability facilitates integration with standard analysis platforms and containerization of the pipeline to enable large-scale, reproducible analyses. The modular design also allows for seamless extensions, such as incorporating deep learning-based segmentation or parcellation tools. Furthermore, MEPrep’s inclusion of both preICA and ME-ICA enables comparisons with legacy pipelines and allows researchers to harmonize across datasets and to develop novel denoising pipelines and preprocessing strategies. Finally, MEPrep’s transparent and user-friendly interface lowers the barrier for adopting multi-echo fMRI.

### 4.2 Addressing the limitations of tedana and ME-ICA

ME-ICA represents a major advance in multi-echo denoising (Cohen et al., 2017; DuPre et al., 2016, 2021; Evans et al., 2015; Heunis et al., 2021; Kundu et al., 2012, 2015, 2017; Lombardo et al., 2016; Lynch et al., 2020; Olafsson et al., 2015; Steel et al., 2022; Waltmann et al., 2019), yet its implementation within tedana has both practical and algorithmic limitations. From a practical standpoint, tedana is not fully integrated with common preprocessing pipelines such as fMRIPrep, lacks native BIDS support, and lacks surface-based outputs. This requires custom workflows that reduce reproducibility and hinder scalability in multi-site studies. From an algorithmic perspective, ME-ICA performs ICA on the optimally combined echo data, which assumes that any echo-specific noise has been minimized (Beckmann & Smith, 2004; Hyvarinen, 1999). In practice, this assumption often fails, especially when artifacts exhibit echo-dependent properties such as slice-level motion, ghosting, or coil drift. These effects can distort ICA decomposition and impair the accuracy of component classification.

Our early-stage denoising strategy, preICA, addresses these issues by applying ICA independently to each echo before combination. This detects echo-specific variance for targeted removal of non-BOLD signals. Additionally, preICA employs probabilistic ICA with Bayesian dimensionality estimation, making component estimation more robust to violations of independence (Beckmann & Smith, 2004). Denoising at the echo level helps maintain the integrity of the multi-echo signal model used in subsequent processing steps.

### 4.3 Sequential ICA and signal complexity

The application of two sequential ICA stages, preICA on individual echoes followed by ME-ICA on the optimally combined data, raises a legitimate concern regarding potential overfitting or excessive smoothing. However, both theoretical considerations and our empirical findings indicate that the dual-ICA strategy enhances, rather than degrades, signal complexity. The two ICA stages operate at different temporal and informational scales. preICA targets millisecond-scale, echo-specific artifacts such as within-slice motion or deviations from exponential decay. ME-ICA, by contrast, operates at the TR scale and targets temporally structured noise such as low-frequency drifts and physiological fluctuations. These stages are complementary rather than redundant, i.e. preICA removes noise orthogonal to that detected by ME-ICA. Our findings support this interpretation. Shannon entropy, a measure sensitive to the complexity and richness of the signal, increased following the full MEPrep pipeline. While aggressive denoising pipelines may inflate tSNR at the expense of entropy, MEPrep improved both, suggesting selective removal of low-complexity noise (e.g., motion or scanner drift) without eroding fine-grained structure in the BOLD signal (McDonough & Nashiro, 2014; Zuo et al., 2014).

Moreover, preICA improves ME-ICA performance in two distinct ways. First, by denoising each echo, it enhances the signal quality of the optimally combined time series used for ICA decomposition, leading to more accurate component separation. Second, ME-ICA’s component classification algorithm, based on the TE-dependence of component time courses, relies on the fidelity of the individual echo time series. Because preICA enhances the quality of these echo-specific time courses, it improves the reliability of ME-ICA’s kappa and rho metrics, which guide classification of components as BOLD or non-BOLD. Thus, preICA contributes to both improved component decomposition and more accurate classification.

### 4.4 Limitations and future directions

MEPrep has several limitations. First, our evaluations were based on a single high-quality dataset. While this allowed us to conduct controlled comparisons across preprocessing methods, generalization to more heterogeneous datasets, such as those with fewer echoes, lower SNR, or scanner variability, needs to be tested. Second, ICA-based methods inherently depend on the stability of the component decomposition. While preICA and ME-ICA were robust across the present sample, convergence failures or component misclassification may occur in lower-quality or short-duration runs (Daubechies et al., 2009; McKeown et al., 1998; Power et al., 2014). Third, the two-step denoising architecture introduces additional computational overhead. Although MEPrep supports parallelization to mitigate this, future development will focus on optimizing run time for larger-scale datasets. Finally, although our results suggest that sequential ICA denoising does not result in over-smoothing, it remains possible that in some datasets, particularly those with low complexity or high noise, this could still pose a risk. However, our entropy analyses indicate that, at least in the current dataset, two-step ICA enhances rather than suppresses meaningful signal variability. We encourage future studies to explore this trade-off further using task-evoked designs and datasets with differing noise characteristics. In addition to addressing some of its limitations, we are exploring the integration of other denoising strategies, such as NORDIC (Chan et al., 2024; Vizioli et al., 2021) and the integration of accelerated anatomical segmentation tools such as FastSurfer (Henschel et al., 2020) to reduce preprocessing time.

## 5 Conclusion

MEPrep is a robust, flexible, and reproducible pipeline for preprocessing multi-echo fMRI data. Its integration of echo-specific preICA denoising, optimal combination, and ME-ICA produces substantial improvements in signal fidelity, reproducibility, and processing efficiency. The pipeline is BIDS-compliant, supports surface and volume-based outputs, and integrates seamlessly into widely used workflows. These attributes make MEPrep well-suited for a broad range of neuroimaging studies, from basic mechanistic work to clinical applications in motion-prone populations. By resolving longstanding challenges in multi-echo preprocessing, MEPrep enables standardized, high-quality analysis of multi-echo fMRI.

## Supplementary Material

Supplementary Material

## Data Availability

Our *MEPrep* pipeline is publicly available on Docker Hub. Users can download and install MEPrep on a Docker-enabled system by running a command under Docker: “docker pull zswang2020/meprep_final:latest”. In addition to the Docker image, the full *MEPrep* source code and documentation have been made available in the GitHub repository at https://github.com/zswang-brainimaging/MEPrep to facilitate extension, community contributions, and integration into other neuroimaging workflows. A quick user guide on how to install and use MEPrep is available in the Supplementary Methods. The readers can test this pipeline using the same dataset as we used, the Siemens dataset (Multi-echo Cambridge), which can be downloaded from https://openfmri.org/dataset/ds000258/.
