# Supplementary material for: MEPrep: A robust pipeline for multi-echo fMRI denoising and preprocessing
Source: Imaging Neurosci (Camb). 2026 Apr 6;4:IMAG.a.1198. doi: 10.1162/IMAG.a.1198 (PMC13055012; doi:10.1162/IMAG.a.1198)

MEPrep: A Robust Pipeline for Multi-Echo fMRI Denoising and Preprocessing

Zhishun Wang, Feng Liu, Rachel Marsh, Gaurav H. Patel, Jack Grinband

SUPPLEMENTAL MATERIALS

Supplemental Methods

The Framework and Implementation of *MEPrep*

The *MEPrep* pipeline uses several platforms and packages including Nipype [1], *fMRIPrep* [2], *tedana*[3], FSL[4, 5], freesurfer[6, 7], ANTs[8], and ICA-AROMA[9], which were integrated, with minimal modification, into native *fMRIPrep* code to facilitate continual software updates. Specifically, we developed new Nipype-based Python scripts to enhance the original fMRIPrep workflows and interfaces, enabling comprehensive handling of multi-echo data within our MEPrep pipeline. These scripts implement novel functionalities and algorithms, including a pre-denoising procedure termed preICA, which leverages probabilistic ICA (PICA) from MELODIC[4], to significantly improve T2*-weighted exponential model fitting. Additionally, we integrated the multi-echo independent component analysis (ME-ICA) functionality provided by the tedana toolkit, further advancing MEPrep's denoising capabilities and overall performance.

To integrate the preICA algorithm and functionalities of the tedana package effectively into the MEPrep pipeline, as illustrated in Figure 1-B and Supplemental Figure 1, we extended the existing fMRIPrep workflow code (t2s.py) by creating a new script, meprep_t2s.py. This extension allowed us to seamlessly incorporate interfaces for preICA and invoke tedana for performing optimal echo combination and ME-ICA. To implement the preICA algorithm comprehensively, we developed several new Nipype interfaces, including preICA_register.py, which registers ICA components to the

standard template space, and dedicated preICA scripts such as preICA_scme.py, designed specifically for executing preICA on spatially concatenated multi-echo data along the Z-axis.

Furthermore, we modified multiple original fMRIPrep scripts to integrate these new functionalities and ensure smooth output handling. These modifications include updates to parser.py for managing pipeline input arguments; workflow management scripts such as base.py, resampling.py, outputs.py, and confounds.py for robustly controlling multi-echo BOLD workflows; and renaming and extending core workflow scripts—t2s.py to meprep_t2s.py, and multiecho.py to meprep_multiecho.py—to link existing workflow components directly to our new preICA implementation. Together, these developments significantly enhance MEPrep's capabilities, streamlining multi-echo preprocessing and improving overall data quality and pipeline performance.

<u>New Nipype codes for extended workflows</u>: meprep_t2s.py, a modified version of t2s.py from the original *fMRIPrep*, is a workflow code to warp the new pre-denoising implementations (interfaces) that we have added based on three ways of running ICA on the inputted raw BIDS data, and *tedana* ME-ICA interfaces. base.py, a modified version of base.py from the original *fMRIPrep*, is a workflow code to handle bold data with adapting to our new code for the implementations of new functions: 1) linking the lower level of workflows such as slice timing correction and motion realignment procedures to prepare data for the pre-denoising procedures; 2) linking the upper level of workflows such as resampling and outputting procedures. resampling.py and outputs.py, modified version of the original code from *fMRIPrep*, to resample and output the new data generated by *MEPrep* including ME-ICA-denoised data and T_2^* model fitting performance data, residual and variance.

<u>New Nipype codes for extended interfaces</u>: The Python script meprep_multiecho.py is an enhanced version modified from the original multiecho.py script provided by fMRIPrep. This updated script integrates the ME-ICA interfaces by directly invoking tedana.py from the tedana software package. In addition, new Nipype interfaces have been developed to support the preprocessing steps required for

preICA: specifically, preICA_register.py, which spatially registers input ICA components to MNI standard space, and preICA_apply_mask.py, which applies pipeline-generated masks to the raw BOLD data. Further, a dedicated Nipype interface, preICA_scme_interface.py, has been created to execute the preICA procedure. This interface performs probabilistic ICA (PICA) denoising on the masked BOLD data prior to T2* model fitting and echo combination. Users can flexibly choose between running PICA on spatially echo-concatenated data or independently on each echo by directly calling FSL's Melodic tool. Collectively, these developments streamline and optimize the preprocessing workflow, significantly enhancing the denoising performance and data quality in multi-echo fMRI analyses.

New Nipypecode for environmental configuration*:* The scripts parser.py and config.py, modified from their original versions in fMRIPrep, incorporate additional configuration options to support the enhanced functionality introduced by MEPrep.

MEPrep User Guide for Preprocessing Multi-echo fMRI Data

Installation

Our MEPrep is Docker-based pipeline. We have made it available to install into users' Docker sever by running the following command:

```
docker pull zswang2020/meprep_final:latest
```

Running MEPrep

Docker Standalone Mode:

Copy and paste the following lines up to ##END into a text editor, replace the required folder names with your own, and save it as a Linux shell script file named "run_meprep.sh". In a Linux terminal, run "bash run_meprep.sh":

```
#!/bin/bash

Input_Dir=/your/path/to/BIDS_Data_Folder_name

Output_Dir=/your/path/to/MEPrep_Output_Folder_Name

Work_Dir=/your/path/to/work_folder_name

Freesurfer_license_Dir=/your/path/to/freesurfer_license_folder

Predenoising_choice=preICA_scme   ## set it to Raw if choosing no pre-denoising

MEPrep_Image=zswang2020/meprep_final:latest

## replace the participant ID of 08999 with your participant ID

## run the Docker image:

docker run -v $Input_Dir:/input  -v  $Output_Dir:/output -v $Work_Dir:/work  -v
$Freesurfer_license_Dir:/fs_license_dir -it -d  $MEPrep_Image  /input  /output  participant  --
participant-label 08999  --tedana-choice Both --preDenoising-choice $Predenoising_choice  --cifti-
output 91k --output-spaces MNI152NLin6Asym:res-2 MNI152NLin2009cAsym:res-native T1w -w
/work  --write-graph --output-layout legacy --fs-license-file /fs_license_dir/license.txt

##END
```

Docker Swarm Mode:

Copy and paste the following lines up to ##END into a text editor, replace the required folder names with your own, and save it as a Linux shell script file named “run_meprep_swarm.sh”. In a Linux terminal, run “bash run_meprep_swarm.sh”:

```
#!/bin/bash

Input_Dir=/your/path/to/BIDS_Data_Folder_name

Output_Dir=/your/path/to/MEPrep_Output_Folder_Name    ## create if not existing before running

Work_Dir=/your/path/to/work_folder_name   ## create if not existing before running
```

```
Freesurfer_license_Dir=/your/path/to/freesurfer_license_folder

Predenoising_choice=preICA_scme   ## set it to Raw if choosing no pre-denoising

MEPrep_Image=zswang2020/meprep_final:latest

Swarm_service_name=MEPrep_job_name

Docker_node_host=your_Docker_swarm_node_hostname

## replace the participant ID of 08999 with your participant ID

## run the Docker service:

nohup docker service create --name $Swarm_service_name  --replicas 1 --reserve-cpu 8 --reserve-memory 16g --mode replicated --restart-condition  none  --constraint "node.hostname==$Docker_node_host"  --mount type=bind,source=$Input_Dir,destination=/input,readonly=true --mount type=bind,source=$Output_Dir,destination=/output --mount type=bind,source=$Work_Dir,destination=/work --mount type=bind,source=$Freesurfer_license_Dir/license.txt,destination=/opt/freesurfer/license.txt,readonly=true  $MEPrep_Image  /input /output -w /work participant --participant-label 08999 --tedana-choice Both --preDenoising-choice $Predenoising_choice --cifti-output 91k --write-graph  --output-spaces MNI152NLin6Asym:res-2 MNI152NLin2009cAsym:res-native T1w

##END
```

Multi-echo fMRI Dataset for Pipeline Validations

We used an open dataset downloaded from openfmri.org website with a dataset number ds000258. We used a sub-set of the data acquired from 32 healthy participants (mean age, 33±13 years, about half males and half females). One run and four-echo resting state fMRI data was acquired from each of the participants. This dataset was acquired on Siemens Trio 3T MRI Scanner and a 32-channel receive-only head coil (Siemens Medical Solutions). Functional images were acquired with anmulti-echo EPI sequence with online reconstruction [repetition time (TR) = 2.47 s; flip angle = 78°; matrix size = 64 × 64; in-plane resolution = 3.75 mm; field of view (FOV) = 240 mm; 32 oblique slices, alternating slice acquisition slice thickness of 3.75 mm with 10% gap; iPAT factor, 3; bandwidth (BW) = 1,698 Hz/pixel; TE = 12, 28, 44, and 60 ms] (9). Anatomical images were acquired using a T1-weighted

magnetization prepared rapid gradient echo (MPRAGE) sequence [FOV = 176 × 240 mm; 1-mm isotropic resolution; inversion time (TI) = 1,100 ms].

Output Data Types Generated by *MEPrep*

The pipeline is designed to generate four types of preprocessed image files in both NIfTI and CIFTI formats, each type accompanied by corresponding confound outputs. These output types consist of OptCom, ME-ICA, pOptCom, and pME-ICA. For clarity, Supplemental Figure 2 provides a concrete example of these output data types generated by running *MEPrep* pipeline Docker container, 'zswang2020/meprep_final:latest' on a subject, sub-04570, from the Siemens dataset.

In Supplemental Figure 2, Panel A-1 showcases two types of preprocessed BOLD CIFTI files and BOLD NIfTI files in standard and native spaces, each paired with corresponding confound files. These files are identified by file names with prefixes 'optcom' and 'optcomDenoised' (ME-ICA type, named in accordance with the tedana workflow naming convention), respectively. They were generated by executing the *MEPrep* command depicted in Panel A-2 without including preICA (setting preDenoising-choice = 'Raw').

In contrast, Panel B-1 illustrates a single type of preprocessed BOLD CIFTI files and BOLD NIfTI files in standard and native spaces, each accompanied by corresponding confound files. These files were produced by executing the *fMRIPrep* command depicted in Panel B-2.

Executing the *MEPrep* command depicted in Panel A-3 with preICA inclusion (setting preDenoising-choice = 'preICA_scme' or 'preICA_se') yields another pair of output types (pOptCom and pME-ICA) following the same file naming convention as shown in Panel A-1. However, this set of data is stored in a distinct folder, labeled 'meprep_preICA_scme' or 'meprep_preICA_se', unlike the previous set of data generated with preDenoising-choice = 'Raw', which resides in the 'meprep_Raw' folder.

REFERENCES

1. Gorgolewski, K. et al. Nipype: a flexible, lightweight and extensible neuroimaging data processing framework in python. *Front Neuroinform* **5**, 13 (2011).
2. Esteban, O. et al. fMRIPrep: a robust preprocessing pipeline for functional MRI. *Nat Methods* **16**, 111-116 (2019).
3. DuPre, E.M., Salo, T., Ahmed, Z., Bandettini, P. A., Bottenhorn, K. L., Caballero-Gaudes, C., Dowdle, L. T., Gonzalez-Castillo, J., Heunis, S., Kundu, P., Laird, A. R., Markello, R., Markiewicz, C. J., Moia, S., Staden, I., Teves, J. B., Uruñuela, E., Vaziri-Pashkam, M., Whitaker, K., & Handwerker, D. A. TE-dependent analysis of multi-echo fMRI with tedana. *Journal of Open Source Software* **6** 1-5 (2021).
4. Beckmann, C.F. & Smith, S.M. Probabilistic independent component analysis for functional magnetic resonance imaging. *IEEE Trans Med Imaging* **23**, 137-152 (2004).
5. Jenkinson, M., Beckmann, C.F., Behrens, T.E., Woolrich, M.W. & Smith, S.M. Fsl. *Neuroimage* **62**, 782-790 (2012).
6. Fischl, B., Sereno, M.I. & Dale, A.M. Cortical surface-based analysis. II: Inflation, flattening, and a surface-based coordinate system. *Neuroimage* **9**, 195-207 (1999).
7. Dale, A.M., Fischl, B. & Sereno, M.I. Cortical surface-based analysis. I. Segmentation and surface reconstruction. *Neuroimage* **9**, 179-194 (1999).
8. Tustison, N.J. et al. Large-scale evaluation of ANTs and FreeSurfer cortical thickness measurements. *Neuroimage* **99**, 166-179 (2014).
9. Pruim, R.H.R. et al. ICA-AROMA: A robust ICA-based strategy for removing motion artifacts from fMRI data. *Neuroimage* **112**, 267-277 (2015).

Supplemental Figure 1

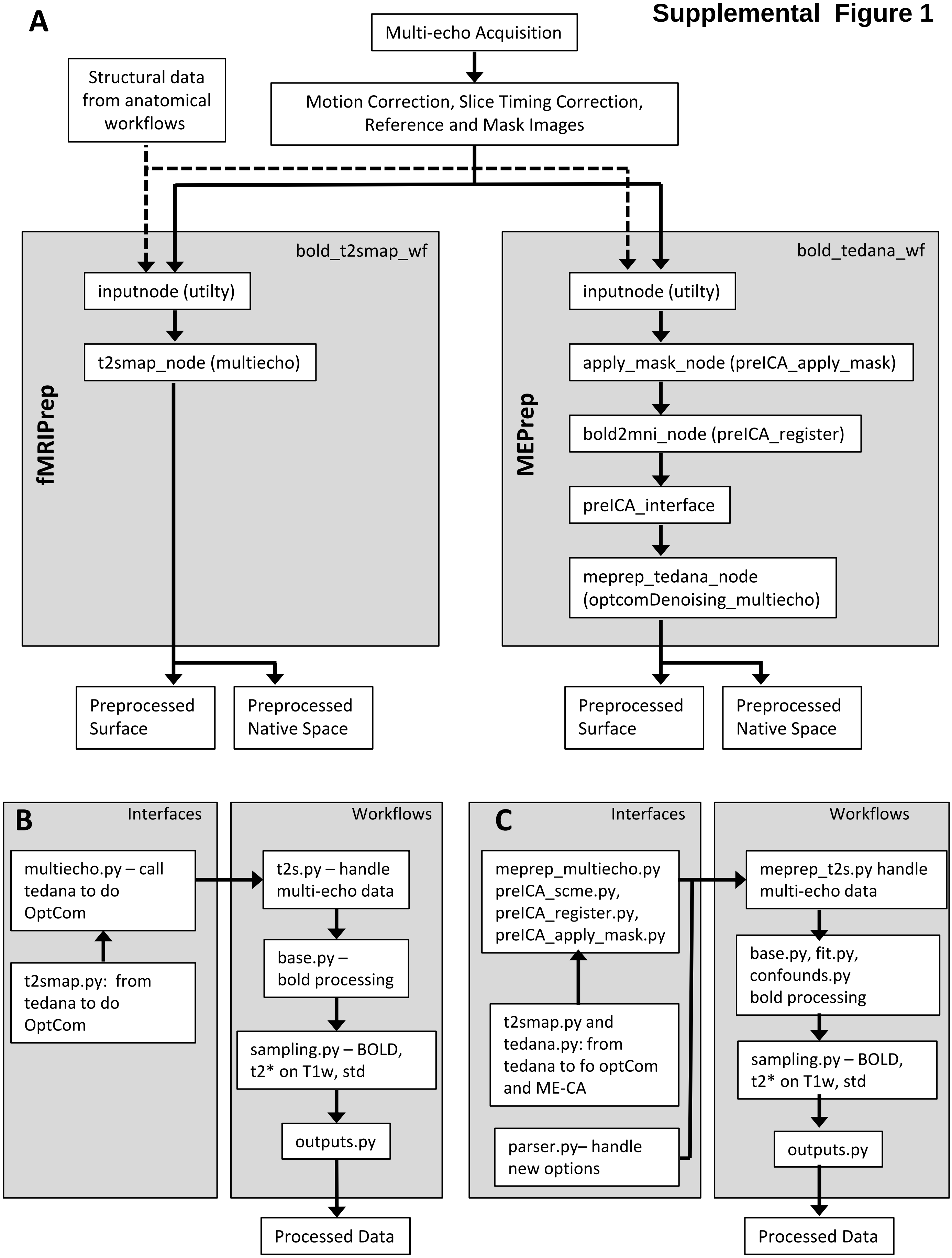

Supplemental Figure 2

A.

```
brain@brainVB:~$ docker run -v $PWD:/Work -it --rm zswang2020/meprep_final:latest /Work/bids_medata /Work/meprep_out participan
t --participant-label 04570 --preDenoising-choice Raw --tedana-choice Both  --output-layout legacy --cifti-output 91k --output-
spaces MNI152NLin6Asym:res-2 MNI152NLin2009cAsym:res-native -w /Work/work_dir --fs-license-file /Work/license.txt
```

B.

```
brain@brainVB:~$ docker run -v $PWD:/Work -it --rm zswang2020/meprep_final:latest /Work/bids_medata /Work/meprep_out participan
t --participant-label 04570 --preDenoising-choice preICA_scme  --tedana-choice Both  --output-layout legacy --cifti-output 91k
--output-spaces MNI152NLin6Asym:res-2 MNI152NLin2009cAsym:res-native -w /Work/work_dir --fs-license-file /Work/license.txt
```

C.

```
brain@brainVB:~$ docker run -v $PWD:/Work -it --rm nipreps/fmriprep:23.1.4 /Work/bids_medata /Work/fmriprep_out participant --p
articipant-label 04570 --output-layout legacy --cifti-output 91k --output-spaces MNI152NLin6Asym:res-2 MNI152NLin2009cAsym:res-
native -w /Work/work_dir --fs-license-file /Work/license.txt
```

D. MEPrep Outputs

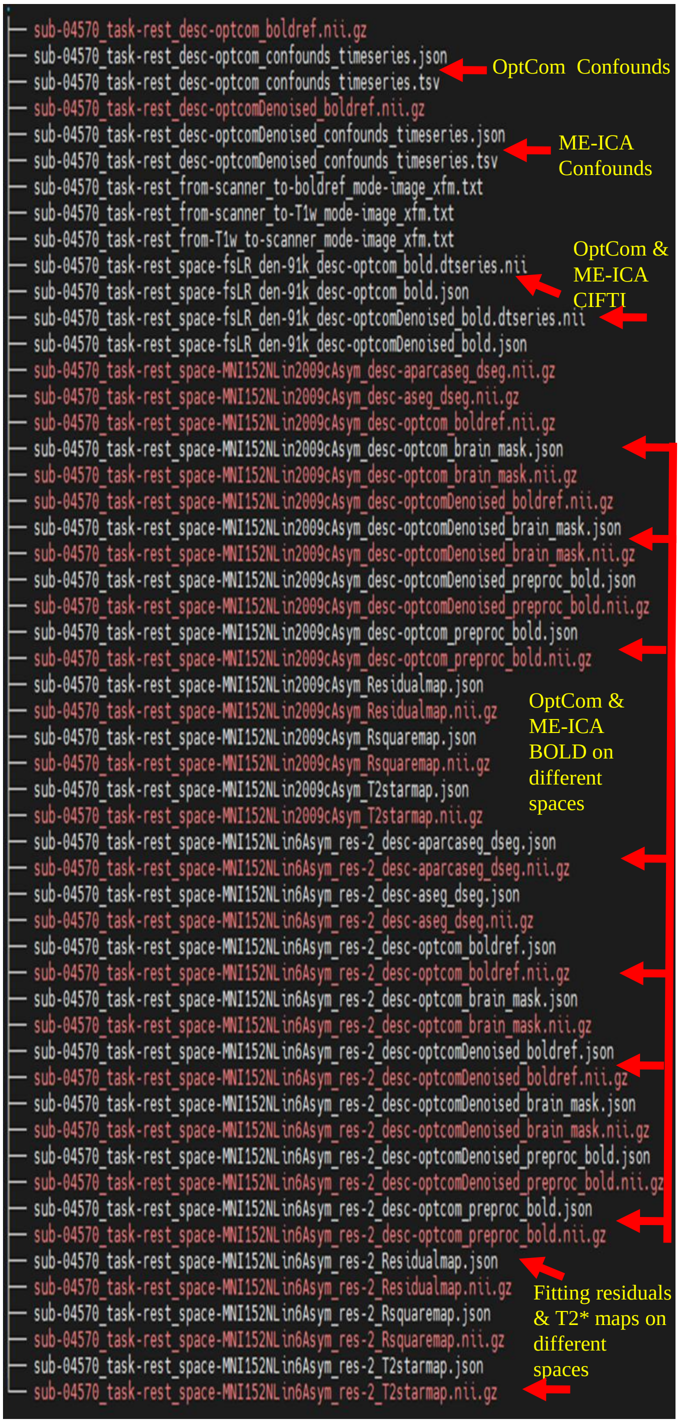

E. fMRIPrep Outputs

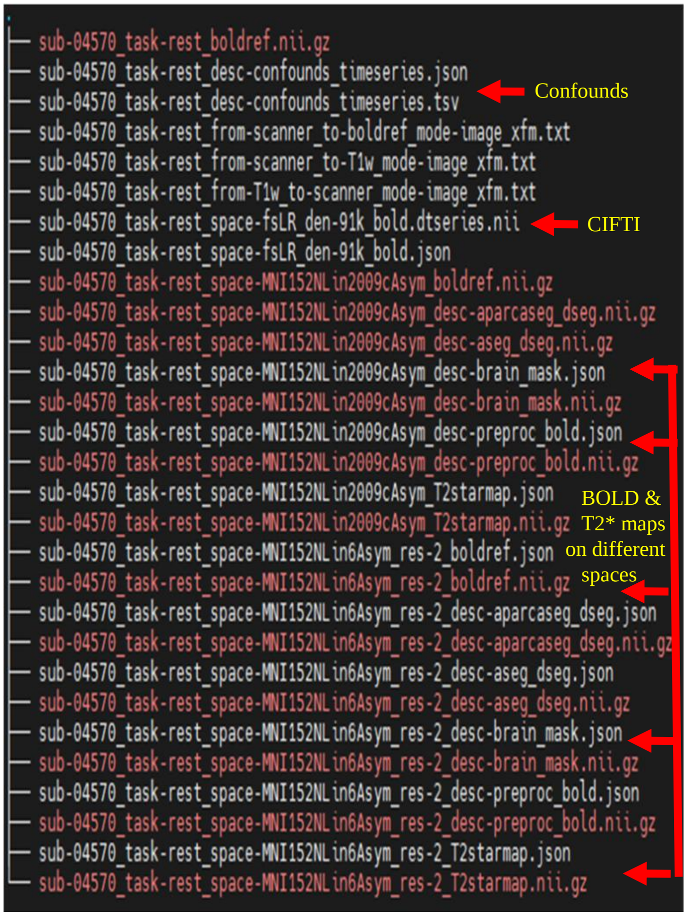

Figure 2. (A) The Docker command of MEPrep that was run to generate the outputs (as shown in D) of OptCom & ME-ICA without preICA on standard and native spaces, stored in meprep_Raw folder. **(B)** The Docker command of MEPrep that was run to generate the outputs (as shown in D) of OptCom & ME-ICA with preICA on standard and native spaces, stored in meprep_preICA folder. **(C)** The Docker command of fMRIPrep that was run to generate the outputs (as shown in E) of OptCom on standard and native spaces, stored in fmriprep folder.

Supplemental Figure 3

A) Reliability of Functional Connectome of MEPrep-Processed BOLD Data on Networks-32 Atlas

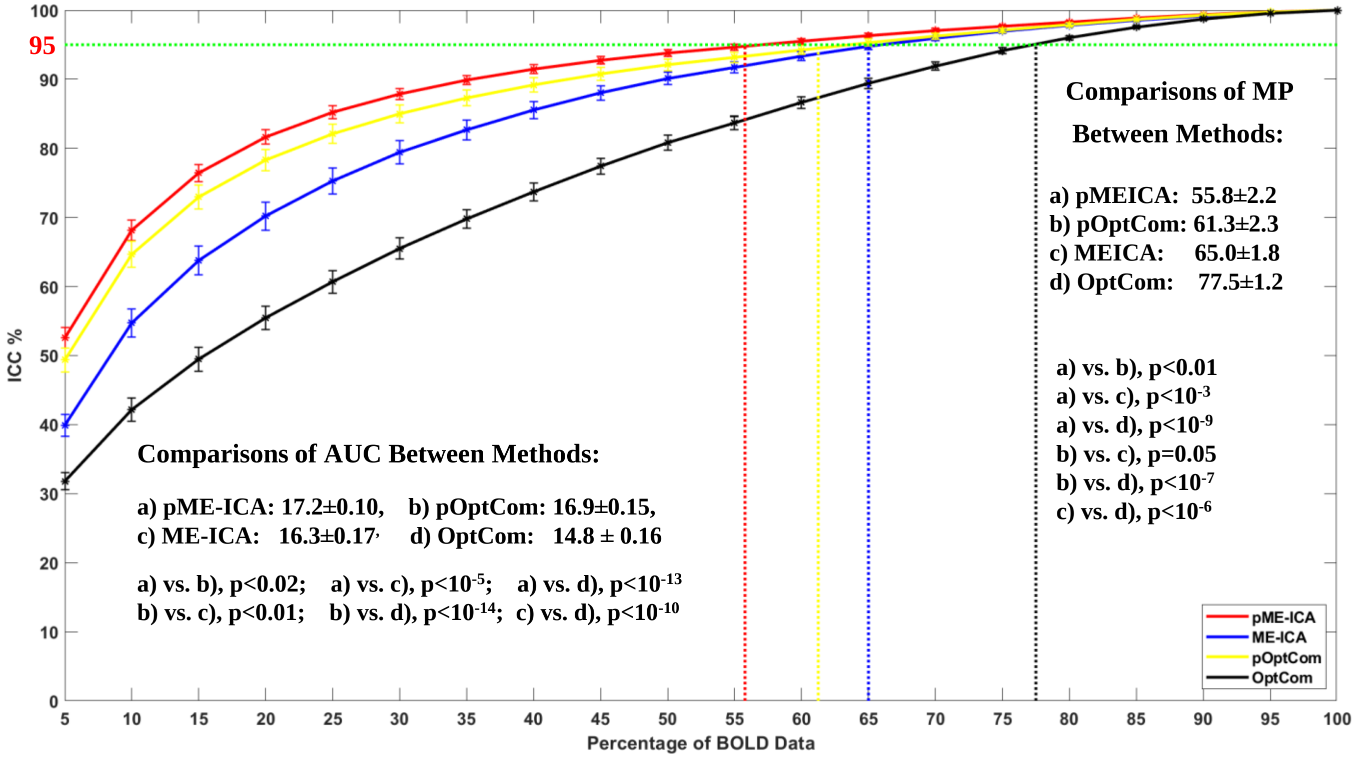

B) Reliability of Functional Connectome of MEPrep-Processed BOLD Data on Networks-268 Atlas

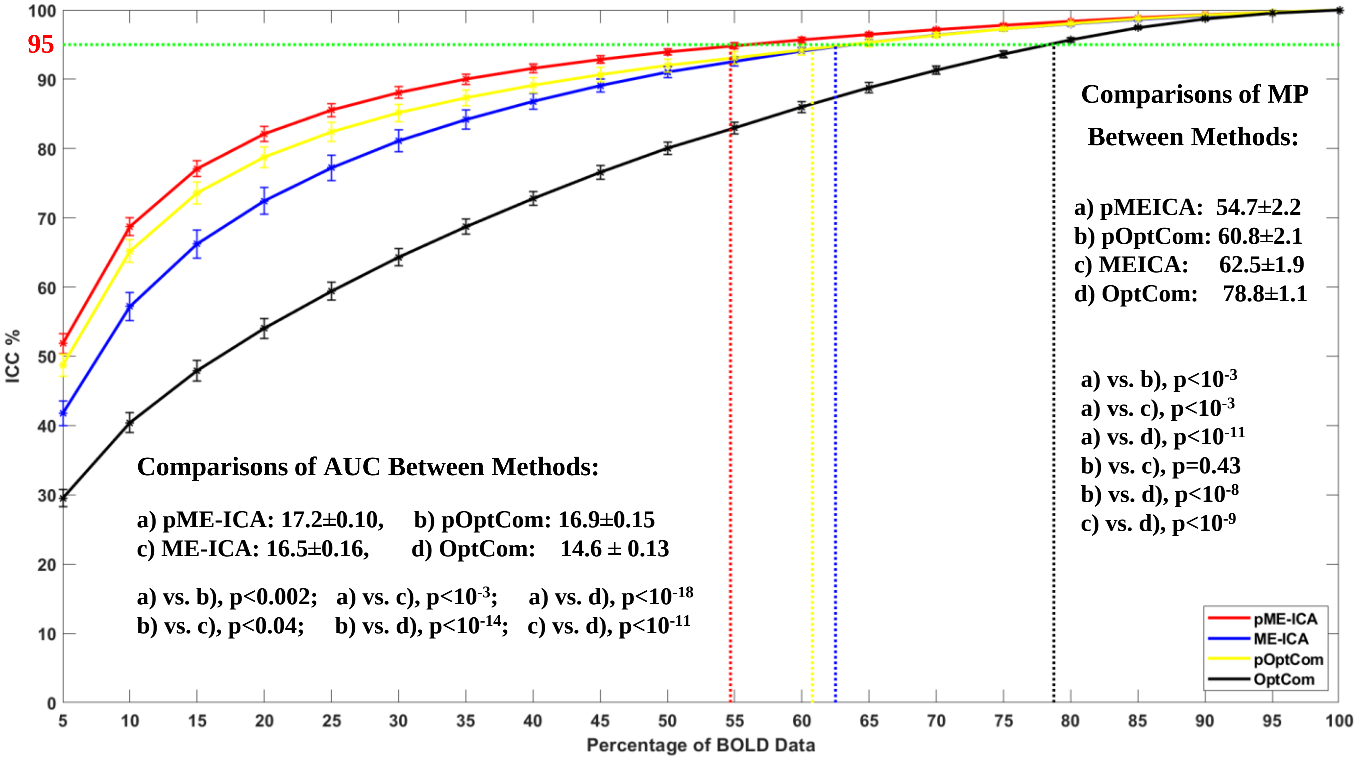

Supplement: Supplementary Material [file IMAG.a.1198_supp.pdf]
